# Frameless Stereotactic Radiosurgery With Linear Accelerator (LINAC)-Based Technology for Brain Metastases: Outcomes Analysis in 141 Patients

**DOI:** 10.7759/cureus.15475

**Published:** 2021-06-06

**Authors:** Aisin Ibrahim, Bernard Fortin, Alexis Bujold, Nader Kaouam, Alma Sylvestre, Christian Boukaram

**Affiliations:** 1 Department of Diagnostic Radiology, McGill University, Montréal, CAN; 2 Department of Radiation Oncology, Maisonneuve-Rosemont Hospital, Université de Montréal, Montréal, CAN

**Keywords:** frameless stereotactic radiosurgery, radionecrosis, linac based srs, brain stereotactic radiosurgery, brain metastasis, treatment of brain metastases

## Abstract

Objectives

Brain metastases (BM) are the most common intracranial tumors in adults. Surgery and frame-based stereotactic radiosurgery (SRS) are well-described treatment options. Frameless SRS is an emerging BM treatment option offering fewer side effects. The aim of this study was to describe the therapeutic outcomes and toxicity of frameless SRS with linear accelerator (LINAC)-based technology for BM treatment in our institution.

Materials and methods

We performed a retrospective study including all adult patients treated with frameless SRS with LINAC-based technology for BM between October 2010 and July 2016. Patients were followed routinely with MRI scans at three-month intervals. Primary endpoints were progression-free survival, local control, overall survival, and toxicity related to the treatment. All survival times were computed with the Kaplan-Meier method. All cumulative incidences were computed using competing risk analyses.

Results

A total of 194 metastatic lesions in 141 patients were treated in a 69-month interval. At the time of analysis, 33 patients were still alive, with a median follow-up time of 25.1 months. The overall median survival was 8.7 months. The median progression-free survival was 5.3 months. Local recurrence as a first event was 25% and 38% at one and two years, respectively, while distant brain recurrence as a first event was 18% and 21%. Death before any brain event occurred in 31% of patients. The cumulative incidence of radiation necrosis as a first brain event was 2% at one and two years.

Conclusions

The treatment of BM with LINAC-based frameless SRS in our institution had an overall and progression-free survival comparable with the literature for frameless SRS and for conventional frame-based SRS while being less invasive and more comfortable for the patient. In our study, frameless SRS with LINAC technology seems to be safe for BM treatment with minimal rates of radiation necrosis.

## Introduction

Brain metastases (BM) are the most common intracranial tumors in adults. Between 20% and 40% of cancer patients develop BM for which a variety of therapeutic options are available, including surgery and radiotherapy [1]. Stereotactic radiosurgery (SRS) has been established as a favorable option in the management of BM [1]. SRS can be used with or without prior surgical resection (Sx) or whole-brain radiation therapy (WBRT) [1-2].

SRS is a high-precision technique that delivers a significant dose of radiation to a localized volume of an organ while minimizing radiation to healthy tissue surrounding it [3]. Conventional radiosurgery was first described in the form of Gamma Knife® (Elekta, Stockholm, Sweden) by Dr. Lars Leksell in 1951. This technique required the use of a rigid head frame for the immobilization of the patient in order to target a precise location in the brain [4]. Although this invasive system provides a high degree of accuracy, it is associated with multiple disadvantages for the patient like pain and anxiety. Also, the rigid head frame requires the presence of a neurosurgeon for frame installation [5-6].

There is now a variety of frameless SRS systems using linear particle accelerator (LINAC) technology that do not require a surgical rigid frame installation while providing better conformity to odd-shaped lesions and sparing of eloquent regions [7]. The aim of this study was to document the therapeutic outcomes and toxicity of frameless SRS with LINAC-based technology for BM in our institution and compare our results with the literature on BM treatment.

## Materials and methods

Patient selection 

This is a retrospective study performed at Maisonneuve-Rosemont Hospital between October 2010 and July 2016. We included all adult patients who underwent frameless SRS for BM with good Recursive Partitioning Analysis (RPA) and Graded Prognostic Assessment (GPA) scores [8-9]. Other inclusion criteria were oligometastatic brain disease (<5 metastasis), BM largest diameter <4 cm, documented primary cancer with a pathology report, controlled extra-cranial disease, and a good Karnofsky performance status (KPS). Patients were assessed by a multidisciplinary team including neurosurgeons, oncologists, and radiation oncologists. 

Non-invasive SRS system and procedures

**Figure 1:**
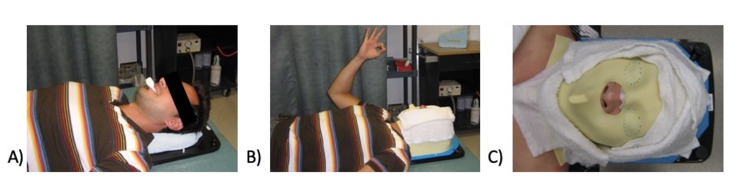
Patient immobilization and frameless SRS mask molding technique (A) A mouthpiece of thermoplastic aquaplast 5 mm is softened and molded to the patient’s mouth by slightly biting with his incisors. (B) Molding of the mask with the mouthpiece in place. (C) Mask left cooling on the patient for about 15 minutes, drawing of the eyes’ limits on the mask, which are later cut out. SRS: stereotactic radiosurgery

Patients were immobilized with a frameless system using an individualized mask molded with a thermoplastic pellet a few days prior to the intervention. A personalized intra-oral thermoplastic piece was incorporated to provide additional rigidity. The time required to produce these masks was between 30 and 45 minutes. The patient immobilization technique and mask molding is shown in Figures 1A-1C.

Following the mask molding, a computed tomography scan (CT) of the brain with contrast was performed with a reconstructed slice thickness of 1 to 1.5 mm. The images were then fused with recently performed gadolinium-enhanced magnetic resonance imaging (MRI) of the brain. Then, contouring of the lesion was done by a radiation oncologist and a margin of 1 to 3 mm was added to the gross tumor volume (GTV) to obtain the planning targeted volume (PTV). The contour of the target lesions and the organs at risk were reverified by a second radiation oncologist before beginning dosimetry planning.

An Elekta Synergy-S® system was used. The patient’s positioning was verified with an on-board cone-beam CT. A HexaPOD^TM ^(Elekta) table was also used for corrections. This allows for repositioning in all axes including rotations.

Patients were candidates to either receive a single dose of radiation or fractionated radiotherapy. The prescribed dose to the PTV was between 12 and 24 Gy with a 15 Gy median. The final radiation dose was decided according to the location and the tumor’s largest diameter [10-12]. Moreover, the volume of tissue receiving more than 10 Gy (V10) or 12 Gy (V12) was taken into consideration in the final radiation dose, as it is an important predictor of radionecrosis (RN) according to Blonigen et al. [13].

Follow-up and statistical analysis

After radiation treatments, the patients were followed routinely with brain MRI at three-month intervals or as clinically indicated. The endpoints of this study were local control (LC), progression-free survival (PFS), overall survival (OS), and evaluation of toxicity induced by the treatment.

OS was defined as the time from initial treatment until death from any cause. PFS was defined as the time from initial treatment until any new local or distant lesion occurred in the brain. LC was defined as the time from initial treatment until disease progression was documented in the bed of a treated metastasis.

All survival times were computed with the Kaplan-Meier method and cumulative incidences of local recurrence were computed using competing risk analyses given the high likelihood of brain recurrences outside of the treated region and concurrent deaths.

Treatment toxicity included clinical and radiological evaluation. In our study, the definition of RN was mainly radiological. If RN was a possibility raised by the radiologist's report on follow-up MRI, regardless of the timing since SRS treatment, it was tagged as RN.

## Results

A total of 141 patients with 194 lesions were reviewed. Forty percent of the patients were male and the cohort had a median age of 62 years ranging between 37 and 89 years at initial treatment. Nearly all the patients had a good performance status with a KPS ≥ 70 (138 patients, 98%) and/or an RPA ≤ II (138 patients, 98%).

SRS as initial management for BM was performed alone in 71 patients (50%). Twenty-one patients (23%) had frameless SRS at the metastatic site after surgical resection and 38 patients (27%) had SRS after whole-brain radiation therapy (WBRT). A small fraction of our cohort, 11 patients (8%), had prior surgery and WBRT before SRS. Baseline patients characteristics and treatments are listed in Table 1 and Table 2.

**Table 1:** Baseline Patients Demographics Characteristics RPA: Recursive Partitioning Analysis, GPA: Graded Prognostic Assessment, KPS: Karnofsky Performance Status

	[n]	%
Total patients	141	100
Sex		
Male	57	40
Female	84	60
RPA class		
I	24	17
II	114	81
III	3	2
GPA class		
1	11	8
1.5	45	32
2	32	23
2.5	25	18
3	17	12
3.5	8	6
4	3	2
KPS		
60	3	2
70	63	45
80	34	24
90	35	25
100	6	4

**Table 2:** Baseline Patients Treatment Plan SRS: Stereotactic Radiosurgery, WBRT: Whole Brain Radiation Therapy, Sx: Surgical Resection

	[n]	%
Total patients	141	100
Treatment plan		
SRS alone	71	50
WBRT + SRS	38	27
Sx + SRS	21	15
WBRT + Sx + SRS	11	8

A total of 194 lesions were treated. Ninety-nine patients (70%) had a single brain lesion, 34 patients (24%) had two brain lesions, and eight patients (6%) had three brain lesions or more. The mean largest diameter of the treated lesions was 17.84 mm, ranging between 1.00 and 72.00 mm with a median of 15.00 mm. Eighty-seven patients (62%) had BM related to primary lung cancer followed by breast cancer in 19 patients (13%) and colorectal cancer in 11 patients (8%). Treated tumors characteristics and primary sites are summarized in Table 3.

**Table 3:** Treated Tumors Characteristics and Primary Cancer Site BM: Brain Metastases

	[n]	%
Total treated lesions	194	100
Number of BM per patient (%)		
1 BM	99	70
2 BM	34	24
3 or more BM	8	6
BM location		
Frontal	70	36
Parietal	30	15
Temporal	20	10
Cerebellar	41	21
Occipital	22	11
Other	11	6
Primary cancer site		
Lung	87	62
Breast	19	13
Colorectal	11	8
Skin melanoma	9	6
Other	15	11

A single fraction of SRS was given in 94% of the radiation treatments. Fractionated radiosurgery was performed in nine patients (6%) and was delivered in three treatment sessions. The median dose to the PTV was 15 Gy and ranged between 12 and 24 Gy. The median volume of tissue receiving 10 Gy (V10) was 14.17 cm^3^ and the median volume of tissue receiving 12 Gy (V12) was 7.81 cm^3^. The median GTV and PTV were 2.91 cm^3^ and 5.23 cm3, respectively. The median Conformity Index was 1.22 and ranged between 0.81 and 2.84 and the median Paddick’s Conformity Index was 0.82 and ranged between 0.53 and 1.53. Treatment parameters are summarized in Table 4.

**Table 4:** Treatment Parameters SRS: Stereotactic Radiosurgery, GTV: Gross Tumor Volume, PTV: Planning Targeted Volume, V10: Volume of brain tissue receiving more than 10 Gy, V12: Volume of brain tissue receiving more than 12 Gy, CI: Conformity Index, CIPaddick’s: Paddick’s Conformity Index

	Mean	Median	Range
Prescribed SRS dose (Gy)	16.60	15.00	12.00 - 24.00
GTV (cm^3^)	6.20	2.91	0.04 – 126.42
PTV (cm^3^)	10.25	5.23	0.31 – 167,65
V10 (cm^3^)	21.25	14.17	2.70 – 319.87
V12 (cm^3^)	12.67	7.81	1.36 – 223.27
CI	1.25	1.22	0.81 - 2.84
CIPaddick’s	0.82	0,82	0.53 - 1.53

The OS was calculated for 139 patients. Two patients were excluded for invalid time reporting. The median OS for all patients was 8.7 months (CI 95%, 4.7-11.9). At the time of analysis, 33 patients (23%) were still alive and the median potential follow-up time was 25.1 months (CI 95%, 16.3-27.9). Fifty-nine patients (42%) had either local or distant BM and the median PFS time was 5.3 months (CI 95%, 3.8-7.0).

**Figure 2:**
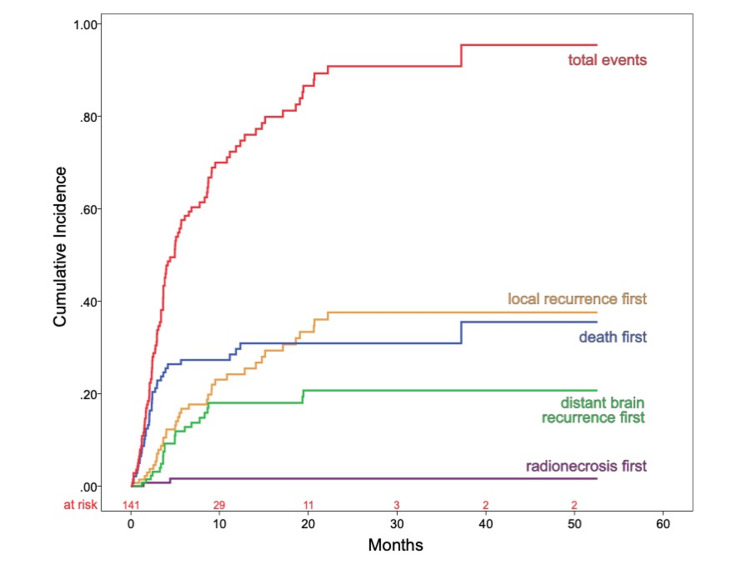
Cumulative Incidence of Radionecrosis, Local Recurrence, Distant Brain Recurrence, and Death as First Event

Local recurrence was seen as the first event in 25% of the patients in the first year and in 38% of the patients in the second year. Distant brain recurrence was seen as the first event in 18% and 21% of the patients, respectively, in the first two years. At one and two years, the cumulative incidence of RN as the first brain event was stable at 2%. Death before any brain event was seen in 39 patients (27%). The total cumulative incidence for death, RN, or any brain recurrence was 74% and 91% at one and two years, respectively (Figure 2).

In our study, only two patients had RN as the most likely brain event; this diagnosis was made on follow-up MRI at 1.4 months and 4.4 months after initial treatment, respectively. Both patients were women with good performance scores (RPA II, KPS ≥ 70, GPA between 1.5 and 2). Both patients had only SRS for their BM with no prior history of WBRT or surgical resection. Both patients were treated with a single fraction SRS of 15 Gy or 18 Gy, respectively. A multivariate analysis was performed to assess for predictors of RN, including sex, RPA, GPA, KPS, prior WBRT or SRS, primary tumor site, number of brain metastases, the use of unfractionated versus fractionated SRS, and the dose prescribed to PTV. Although, unsurprisingly, with only two events, none were found to be statistically significant.

## Discussion

The efficacy of LINAC-based SRS for extra-cranial tumors has been well-documented in the literature for the lungs, liver, and spine [14]. Nowadays, SRS is increasing in popularity for BM treatment and multiple non-invasive frameless LINAC delivery units exist, but the data on their therapeutic impact are still to be gathered. 

This retrospective study reports data on 141 patients treated with frameless LINAC-based SRS for intracranial metastasis from various primary sites. Our objective was to document SRS BM treatment outcome and toxicity in our institution.

At the time of analysis, 33 patients were still alive with a median follow-up time of 25.1 months. Our cohort had an overall median OS of 8.7 months, which is comparable to the Hauswald et al. [15] and Breneman et al. [6] studies that reported a median OS of 9 and 8.1 months, respectively. Minniti et al., however, reported a longer OS of 14.1 months in a 206 patient-based study [2]. The differences between our study and Minniti et al. could be explained by a difference in the patient selection process. Minniti et al. based their study on patients with BM size <3.5 cm, and they excluded all patients previously treated with SRS, WBRT, or surgery. The inclusion criteria in our study were wider and included patients with larger BM, up to 4 cm, and possibly more aggressive cancers since we included patients who had prior surgical resection and/or WBRT. Furthermore, three patients (2%) had a documented KPS score of 60, but they were still included in our study, as they were found to benefit from frameless SRS on clinical evaluation. The median PFS in our study was 5.3 months, which is also comparable to data reported in the literature. A comparison and overview of the prior literature on frameless LINAC-based SRS is reported in Table 5.

**Table 5:** Frameless LINAC-Based SRS: Overview of Prior Studies NR: Not Reported, Pts: Patients, BM: Brain Metastases, LINAC: Linear Particle Accelerator, SRS: Stereotactic Radiosurgery, RPA: Recursive Partitioning Analysis

	Kamath et al. [16]	Breneman et al. [6]	Nath et al. [5]	Minniti et al. [2]	Hauswald et al. [15]	Present study
[n] patients	64	53	65	206	84	141
[n] BM treated	NR	158	204	310	140	194
SRS technique	Frameless LINAC-based SRS	Frameless LINAC-based SRS	Frameless LINAC-based SRS	Frameless LINAC-based SRS	Frameless LINAC-based SRS	Frameless LINAC-based SRS
Prescribed dose	17.5 Gy (median)	18 Gy (median)	18 Gy (median)	18 Gy (mean)	20 Gy (median)	15 Gy (median)
[n] BM per patient (range)	Median: 2 (1–4)	Median: 2 (1–15)	Median: 2 (1–13)	1 : 126 pts	1 : 49 pts	1 : 99 pts
				2 : 56 pts	2–3: 31 pts	2–3: 40 pts
				3 : 24 pts	≥ 4 : 4 pts	≥ 4 : 2 pts
OS (median)	8.7 mo	RPA I: 18 mo	40% at 1 year	14.1 mo	9 mo	8.7 mo
		RPA II-III: 8.1 mo				
PFS (median)	8.1 mo	NR	46% at 1 year of regional control	10 mo	5.3 mo	5.3 mo
Median follow-up time	8.2 mo (overall)	NR	18.1 mo (survived patient)	9.4 mo (overall)	7 mo (overall)	25 mo (survived patient)
			6.2 mo (overall)			
[n] patients alive at time of the analysis (%)	NR	29 (55%)	16 (25%)	91 (44%)	11 (19%)	33 (23%)
[n] radionecrosis (%)	none	2 (3.8%)	1 (1.5%)	12 (5.8%)	1 (1.2%)	2 (1.4%)

The median OS in our study is also similar to the frame-based SRS technique in the literature. A large, prospective, SRS frame-based study by Serizawa et al. [17] reported a median OS at 8.3 months for RPA II class patients (n = 2150). However, to our knowledge, no prospective study has yet compared these two modalities.

Kimmel et al. [18] published a meta-analysis on brain metastasis management and, according to their data, SRS, with or without WBRT, should be the initial treatment for BM. Based on the present study and frameless LINAC-based SRS literature, it is not only possible to use that strategy as initial treatment for BM, but it is also an option associated with acceptable PFS and OS time. When comparing traditional frame-based SRS literature with frameless SRS, non-invasive frameless SRS offers better comfort levels and decreases anxiety for the patient [6]. Frameless SRS also allows for an easier way to deliver fractionated radiotherapy, which could be useful when the brain metastases are large, oddly shaped, or near critical structures such as cranial nerves, deep gray matter, or the brainstem [6,12,19-21]. Furthermore, the use of SRS without WBRT was associated with fewer side effects like hair loss, fatigue, and cognitive dysfunction [22].

As for the adverse events documented in our cohort, two patients (1.4%) developed radiological findings of RN on follow-up MRI. Analysis of the demographic and prognostic parameters for RN was not found to be statistically significant. However, this is expected given only two cases of RN were reported. This is comparable to the rate of RN previously documented in prior studies, stating a range of RN between 1.2% and 5.8% [2,5-6,15]. However, imaging alone remains notoriously inaccurate in diagnosing RN, the gold standard remains a brain biopsy, which was not performed in our patients. Moreover, imaging features of RN may overlap with findings of cancer recurrence or pseudo-progression, therefore overestimating the incidence of RN [23-24]. Although there is a low incidence of RN in our study, a case report published by Hyde C et al. [25] states that fractionated radiotherapy may provide a reduction in the incidence of toxicity, including RN, and it is a safe and well-tolerated method for the treatment of multiple brain metastasis [26].

Our study had some limitations. First, the main limitation is related to its retrospective nature, making follow-up data inherently suboptimal. Second, our patient cohort was heterogeneous, with various prior treatments and primary neoplasms, which limited our analysis. Third, when we first initiated this technique in our center, we only selected patients with good performance status (RPA, GPA, KPS). However, with new data on SRS use for BM management being published, we loosened our inclusion criteria, therefore enlarging our population to more severe diseases.

Overall, the first results of our study seem promising for frameless LINAC-based SRS, and to our knowledge, this study has one of the longest follow-up periods in a retrospective study in the literature.

## Conclusions

The treatment of BM with LINAC-based frameless SRS in our institution had an OS and PFS comparable with the literature for frameless SRS and for conventional frame-based SRS while being less invasive and more comfortable for the patient. In our study, frameless SRS with LINAC technology seems to be safe for BM treatment with minimal rates of radiation necrosis.
